# Bruton’s tyrosine kinase-bearing B cells and microglia in neuromyelitis optica spectrum disorder

**DOI:** 10.1186/s12974-023-02997-2

**Published:** 2023-12-21

**Authors:** Ye Liu, Zhenning Huang, Tian-Xiang Zhang, Bin Han, Guili Yang, Dongmei Jia, Li Yang, Qiang Liu, Alexander Y. L. Lau, Friedemann Paul, Alexei Verkhratsky, Fu-Dong Shi, Chao Zhang

**Affiliations:** 1 Department of Neurology and Institute of Neuroimmunology, Tianjin Medical University General Hospital, 154 Anshan Road, Heping District, Tianjin, 300052 China; 2 Present Address: Center of Neurological Diseases, China National Clinical Research Center for Neurological Diseases, Beijing Tiantan Hospital, Capital Medical University, Beijing, China; 3 Division of Neurology, Department of Medicine and Therapeutics, Faculty of Medicine, The Chinese University of Hong Kong, Shatin, Hong Kong, China; 4 Experimental and Clinical Research Center, Max Delbrueck Center for Molecular Medicine and Charité-Universitaetsmedizin Berlin, Berlin, Germany; 5 NeuroCure Clinical Research Center, Charité-Universitaetsmedizin Berlin, Berlin, Germany; 6 Faculty of Biology, Health and Medicine, University of Manchester, Manchester, M13 9PL UK; 7 Achucarro Centre for Neuroscience, IKERBASQUE, Basque Foundation for Science, 48011 Bilbao, Spain; 8 Department of Stem Cell Biology, State Research Institute Centre for Innovative Medicine, 01102 Vilnius, Lithuania

**Keywords:** Neuromyelitis optica spectrum disorder, B cells, Microglia, Bruton’s tyrosine kinase, Zanubrutinib

## Abstract

**Background:**

Neuromyelitis optica spectrum disorder (NMOSD) is an inflammatory autoimmune disease of the central nervous system that involves B-cell receptor signaling as well as astrocyte–microglia interaction, which both contribute to evolution of NMOSD lesions.

**Main body:**

Through transcriptomic and flow cytometry analyses, we found that Bruton’s tyrosine kinase (BTK), a crucial protein of B-cell receptor was upregulated both in the blood and cerebrospinal fluid of NMOSD patients. Blockade of BTK with zanubrutinib, a highly specific BTK inhibitor, mitigated the activation and maturation of B cells and reduced production of causal aquaporin-4 (AQP4) autoantibodies. In a mouse model of NMO, we found that both BTK and pBTK expression were significantly increased in microglia. Transmission electron microscope scan demonstrated that BTK inhibitor ameliorated demyelination, edema, and axonal injury in NMO mice. In the same mice colocalization of GFAP and Iba-1 immunofluorescence indicated a noticeable increase of astrocytes–microglia interaction, which was alleviated by zanubrutinib. The smart-seq analysis demonstrated that treatment with BTK inhibitor instigated microglial transcriptome changes including downregulation of chemokine-related genes and genes involved in the top 5 biological processes related to cell adhesion and migration, which are likely responsible for the reduced crosstalk of microglia and astrocytes.

**Conclusions:**

Our results show that BTK activity is enhanced both in B cells and microglia and BTK inhibition contributes to the amelioration of NMOSD pathology. These data collectively reveal the mechanism of action of BTK inhibition and corroborate BTK as a viable therapeutic target.

**Supplementary Information:**

The online version contains supplementary material available at 10.1186/s12974-023-02997-2.

## Introduction

Neuromyelitis optica spectrum disorder (NMOSD) is an autoimmune disease of the central nervous system (CNS) that predominantly affects the spinal cord, optic nerves, and area postrema. In ≥ 80% of cases, NMOSD is instigated by anti-aquaporin-4 antibodies (AQP4–IgG), which target and damage AQP4-rich astrocytic endfeet that form glia limitans perivascularis [1–3]. Progressive disability results from frequent and severe relapses of NMOSD attacks. Therefore, long-term preventive immunotherapy is mandatory to slow down the progression of the disease [4]. Therapies aimed at depleting B cells, such as anti-CD20 and anti-CD19 monoclonal antibodies, were effectively used for preventing recurring attacks [5–9]. However, approximately 20% of patients are resistant to B-cell depletion treatment [10, 11]. Optimization of B-cell-targeted treatments without B-cell depletion relies on the precise characterization of B-cell subsets and their corresponding molecular signatures in NMOSD.

B cells contribute to the pathogenesis of NMOSD through antibody production, antigen presentation and production of neurotoxic molecules and pro-inflammatory cytokines [12–14]. First and foremost, B cells are precursors of plasmablasts and plasma cells that produce and release AQP4–IgG [15]. The water channel AQP4 is concentrated at astrocytic endfeet, which form glia limitans perivascularis. Binding of IgG to astrocytic AQP4 triggers complement cascade and secretion of cytokines and chemokines, resulting in AQP4 loss, damage of endfeet as well as astrocytes, and reactive microgliosis [16, 17]. Recent studies demonstrated that B-cell-depleting agents may attenuate reactive gliosis in mouse experimental autoimmune encephalomyelitis (EAE), a model for multiple sclerosis [18, 19]. In addition, some researches also showed that rituximab, a monoclonal antibody targeting the CD20 antigen, was effective in a large proportion of chronic inflammatory demyelinating polyradiculoneuropathy (CIDP) patients [20, 21], indicating that B-cell-targeted intervention could be useful in both the peripheral and CNS demyelinating diseases.

Using the high throughput method of single-cell RNA-sequencing (scRNA-seq), we characterize the diversity of B-cell subpopulations and their dysregulated molecular expression in NMOSD; we further identify activation of Bruton’s tyrosine kinase (BTK) as a critical mediator of B-cell activation in NMOSD. BTK is a nonreceptor tyrosine kinase critical for proper B-cell development and signaling. The activity of BTK is regulated by Src-mediated phosphorylation of the kinase domain at tyrosine 551. This initial phosphorylation event is followed by autophosphorylation at Tyr223. Phosphorylated BTK then associates with the cell membrane through interacting with the pleckstrin homology (PH) domain with phosphatidylinositol 3, 4, 5-triphosphate [22, 23]. As BTK is also expressed in microglia we cauterized its role in microglial reactivity and microglia–astrocyte interactions in a NMO mouse model. Our data establish foundational evidence to explore BTK inhibitors as a potential new therapy for NMOSD.

## Materials and methods

### Patients and healthy individuals

Twenty-eight patients with NMOSD from Tianjin Medical University General Hospital with positive serum AQP4–IgG (by an in-house cell-based assay) and widely varying durations of the disease onset between 18 February 2020 and 1 February 2022 were recruited. All patients met the International Panel for NMO Diagnosis (IPND) 2015 criteria for NMOSD [24]. Clinical data sets were extracted from inpatients case history. Twenty-one healthy volunteers were enrolled as control subjects. Full informed consent was obtained and the work was performed under Research ethics committee approvals IRB2021-KY-134.

Samples from twelve patients with NMOSD during acute attacks (before treatment with steroids or plasmapheresis) (CSF samples, *n* = 4; blood samples, *n* = 6; bone marrow from hipbone, *n* = 2) and five healthy controls (HCs) (blood samples, *n* = 3; bone marrow from hipbone, *n* = 2) were collected for scRNA-seq (Table 1), which included previously published scRNA-seq data of CSF, peripheral blood and bone-marrow samples from patients with NMOSD [13]. In addition, we added one peripheral blood sample from a new treatment-naive patient with NMOSD in an active relapse stage and bone-marrow samples from two HCs.

**Table 1:** Demographics of patients with NMOSD and healthy controls

	single-cell RNA-sequencing		Flow cytometry analysis of BTK/pBTK expression	
	HCs (*n* = 5)	NMOSD (*n* = 12)	HCs (*n* = 16)	NMOSD (*n* = 16)
Female, *n* (%)	5 (100)	11 (91.6)	14 (87.5)	15(93.7)
Age, median (IQR)	46.5 (43.5–54.0)	51.0 (49.5–60.5)	48.5 (31.5–52.5)	59.5 (39.5–63.0)
Age at onset, median (IQR)	n/a	47.5 (40.8–54.0)	n/a	53.7 (35.0–58.7)
Disease duration (years), median (IQR)	n/a	3.5 (0.3–8.5)	n/a	3.8 (2.5–7.6)
EDSS score, median (IQR)	n/a	4.0 (3.5–6.5)	n/a	3.5 (2.5–7.0)
AQP4-ab positive, *n* (%)	n/a	12 (100)	n/a	16 (100)
Treatment before relapses, *n* (%)				
No treatment	n/a	7 (58.3)	n/a	9 (56.25)
Oral corticosteroids		5 (41.7)		7 (43.75)

Data are expressed as median (25th–75th percentiles), unless otherwise specified. All percentages are calculated for patients with available data

*EDSS* expanded disability status scale, *HC* healthy control, *NMOSD* neuromyelitis optica spectrum disorder

Besides that, sixteen NMOSD patients during acute attacks (before treatment) and 16 age- and sex-matched HCs were enlisted to assess BTK and phosphorylated BTK (pBTK) expression in B-cell subpopulations (Table 1). All samples were obtained under informed consent and in accordance with the Institutional Review Board of Tianjin Medical University General Hospital.

### scRNA-seq

#### Single cell acquisition

The samples were processed for scRNA-seq as described previously [13]. In brief, peripheral blood mononuclear cells (PBMCs) were isolated from fresh whole blood or bone marrow via density gradient by Ficoll–Paque and cells in CSF were obtained by centrifugation (2000 rpm × 5 min). Single cell suspensions were blocked with 5 μl of Fc receptor blocking solution (BioLegend, 422302) and thereafter, incubated with 7-AAD Viability Staining Solution (BioLegend, 420403), CD3 (BioLegend, clone OKT3), CD14 (BioLegend, clone 63D3) and CD16 (BioLegend, clone 3G8). B cells were sorted using FACS Aria III (BD Biosciences, San Jose, CA, USA). Sequencing libraries were prepared using 5′ library preparation kits (10 × Genomics, Pleasanton, CA, USA).

#### Quality control for barcode hopping

Using a custom pipeline, overlapping reads present in more than one sample sharing the same cell barcode and unique molecular identifier (UMI) were counted and filtered using a single sample read assignment threshold percentage of 80% as previously described [25].

#### Cell type annotation and differential gene expression analysis

Samples were run on 10 × Genomics that were aligned to the human GRCh38 genome using CellRanger version 3.0.2. B-cell identity types were defined by performing differential expression genes (DEGs) analysis for each cluster. The normalized gene expression profiles for distinct clusters were compared with the remaining cells using Seurat’s FindAllMarkers function (default parameters). The most up-regulated genes, with the highest positive average log fold change, were compared with a custom panel of canonical gene makers spanning several key immune cell types, including naïve B cells, memory B cells, and antibody secreting cells. In addition, to improve specificity, all B cells included in our analyses were required to have reads of CD79A. Heat map generation was performed using the FindMarkers command in Seurat with the Wilcoxon test and the following parameters: *p*-adjusted value cutoff = 0.05 and log FC cutoff = 0.5.

#### Pathway analysis

Pathway analysis was carried out using the ingenuity pathway analysis (IPA) toolkit (v01.12). We reported all pathways that were significant at Benjamini–Hochberg *p* adjusted of 0.05. In addition, upstream regulator analysis and causal network analysis were carried out using the IPA toolkit to identify likely upstream regulators of genes in the data set. The activation state of a pathway or gene was calculated based on differentially expressed genes (*p* adjusted = 0.05), and all pathways/causal networks/master upstream regulators with a *p* adjusted less than 0.05.

### Quantitative RT-PCR validation of selected genes

B cells from NMOSD patients and healthy controls were isolated from PBMCs by magnetic-activated cell sorting techniques according to the protocol provided by Human B-cell Isolation kit II (130-091-151, Miltenyi Biotec, Auburn, CA) and were used for RNA extraction. RNA was reverse transcribed into cDNA and levels of different genes were determined by real-time PCR (RT-PCR). The primers used are listed as follows: *BTK* primers (forward 5′-TTAAGTCAGGACTGAGCACACA-3′; reverse 5′-AGGCGGTAGTGGCTTTTTCA-3′), *LYN* primers (forward 5′-TAAACAGCAAAGGCCAGTTCC-3′; reverse 5′-TTGCCACCTTCATCGCTCTT-3′), *BCL2* primers (forward 5′-TGCTTGGGGACAAAACCCTT-3′; reverse 5′-CCTGGGAAAGTTAGGGGCAG-3′), *PIK3CA* primers (forward 5′-GATGCAGCCATTGACCTGTT-3′; reverse 5′-GCTCGAGCTCACCTCTCAA-3′), *CD40* primers (forward 5′-AGGATGGCAAAGAGAGTCGC-3′; reverse 5′-GCTCATGTCACCCAGCATGT-3′), *JAK1* primers (forward 5′-GCAGTGTGTCTGGGGGAAAT-3′; reverse 5′-GTGCCAAACACAATGGCAGT-3′),

*STAT1* primers (forward 5′-CAGAGCACTCTGGTCAAGCA-3′; reverse 5′-AAACCCAACATACAGGACTTCT-3′) and *BLK* primers (forward 5′-CCCAGCTAAACAAGAGCGGT-3′; reverse 5′-AAGCCACCAAGCCTACCTTTG-3′). Quantitative RT-PCR was performed in triplicate in 96-well plates using a qPCR machine (LC480, Roche) and SYBR Green I Master mixture (4887352001, Roche) to detect amplification products generated using the following thermocycling protocol: initial denaturation at 95 ℃ for 10 min followed by 40 amplification cycles of 95 ℃ for 15 s and 60 ℃ for 1 min, and a final cycle at 25 ℃ for 15 s. Relative quantification of mRNA expression was performed using the comparative cycle method to obtain this ratio: gene of interest/GAPDH. Relative quantification of gene-expression levels was performed using the 2-DDCt method.

#### Flow cytometry

Fluorescence labeling of cells and measurement of total intracellular BTK levels were performed as described previously [26, 27]; the BTK antibodies for total BTK protein came from Cell Signaling Technology (71500). BTK gate settings were based on isotype controls and fluorescence minus one control. For staining of phosphorylated BTK, PBMCs were either unstimulated or stimulated for 30 s with F(ab′)_2_ anti-human IgM (20 μg/ml; Life Technologies, Carlsbad, CA, USA) and followed by fixed with Cytofix for 20 min and permeabilized with Phosflow Perm Buffer III (BD Biosciences), and anti-BTK Phospho (Tyr223) antibody came from Biolegend (601704). Flow cytometric measurements were performed on FACS Aria III (BD Biosciences, San Jose, CA, USA). Data were analyzed using FlowJo software (Version X; TreeStar, Ashland, OR, USA).

### In vitro peripheral blood B-cell experiments

#### B-cell culture and flow cytometry

B cells were isolated from PBMCs by magnetic-activated cell sorting techniques according to the protocol provided by Human B-cell Isolation kit II (130-091-151, Miltenyi Biotec, Auburn, CA) after PBMCs were separated by density centrifugation. The purity of B-cell population was routinely confirmed as > 95% by detecting the proportion of CD19^+^ cells through flow cytometry. B cells were plated in 96-well plates at 2 × 10^5^/well in a total volume of 200 μl per well with or without stimulated by F(ab′)_2_ anti-human IgM (10 μg/ml) and in the presence or absence of BTK inhibitor zanubrutinib (10 nM) for 2 days. Then, B cells were collected for flow cytometry. Fluorescence labeling of cells and measurements of intracellular BTK levels were performed as described above.

#### PBMCs culture and AQP4 antibody determination of the culture supernatants

Following peripheral blood sample were isolated from patients with NMOSD, PBMCs were separated by density centrifugation. PBMCs were plated in U bottom 96-well plates at 3 × 10^5^/well in a total volume of 200 μl per well. These cells were in the presence of IL-21 (100 ng/ml), sCD40L (100 ng/ml), IL-6 (10 ng/ml), TNF-α (10 ng/ml), R848 (2.5 μg/ml) and with or without BTK inhibitor zanubrutinib (10 nM) for 7 days. Supernatants were collected for AQP4–IgG titer detection as previously reported [13, 28].

#### NF-κB nuclear translocation

The association between zanubrutinib and canonical NF-κB signaling was addressed using imaging flow cytometry (Merck Millipore, Billerica, MA, USA). Following the activation of PBMC with LPS (50 ng/ml) for 30 min in the absence or presence of zanubrutinib (10 nM), CD19^+^ B cells were individually interrogated by imaging flow cytometry simultaneously for (a) binding to the PE stained pBTK, and (b) activation status as indicated by the localization of Alexa Fluor 488-labeled NF-κB either in the cytoplasm (for non-activated cells) or the nucleus (for activated cells). The spatial relationship between the NF-κB and nuclear images was measured using the ‘Similarity’ feature in the IDEAS® software as described previously [29]. Briefly, a ‘Morphology’ mask is created to conform to the shape of the nuclear 7-AAD image, The ‘Similarity Score’ (SS) is a log-transformed Pearson’s correlation coefficient between the pixel values of two image pairs, and provides a measure of the degree of nuclear localization of NF-κB by measuring the pixel intensity correlation between the NF-κB images and the 7-AAD images within the masked region. Higher score signifies greater concentration of intensity inside the cell. Cells with internalized signal typically have positive scores, whereas cells with little internalization have negative scores.

### In vivo animal experiments

#### Animals

Thirty wild-type female, 8–10-week-old C57BL/6 mice were purchased from Vital River (Beijing, China) and were housed in the animal facility for 1 week prior to the start of experiments. The mice were randomly divided into 5 groups (*n* = 6 per group). One group underwent intracranial injection surgery with control–IgG as control group, and the others underwent intracranial injection surgery with NMO–IgG. The NMO–IgG recipients were then randomly divided into 4 groups: three groups receiving 5, 10, or 20 mg/kg BTK inhibitor zanubrutinib respectively) and a vehicle group. All of animal procedures were approved by the Animal Experiments Ethical Committee of Tianjin Medical University and performed according to the Revised Guide for the Care and Use of Laboratory Animals.

#### Induction of NMO mouse model and administration of BTK inhibitor zanubrutinib

NMO mouse model was induced as described in previous studies [30, 31]. Mice were anaesthetized with isoflurane and mounted onto a stereotactic frame (RWD Life Science, Shenzhen, Guangdong, China). A burr hole was drilled in the skull 2.5 mm to the right of the bregma under a midline scalp incision. A 30-gauge needle attached to a 50 μl gas-tight glass syringe (Hamilton, Reno, NV, USA) was advanced 3 mm deep to infuse 12 μl mixture of NMO–IgG + human complement (hC) (volume ratio 3:2). The concentration in NMO–IgG was 9.4 mg/ml. Three dosages of zanubrutinib (5, 10, or 20 mg/kg) or vehicle were administered (i.p.) 12 h after modeling. The administration was conducted daily for 5 days. Control–IgG (SP001, Solarbio, Beijing, China) did not bind to live HEK293 cells transfected with mouse or human AQP4 and were delivered by the same intracranial injection surgery with the same volume. In this study, we verified the NMO model through immunofluorescence staining of AQP4, and only data from the mice with loss of AQP4 were included in the analysis.

#### Rotarod test and gait analyses

Rotarod performance was completed using a 5-lane apparatus (Med Associates Inc). Animals were placed on the accelerating rotarod (increasing from 4 to 36 rpm over a 4-min period). The latency to fall on the rotarod was recorded. Each mouse was tested 3 times at 15-min intervals.

The Catwalk XT 10.0 gait analysis system (Noldus Information Technology, Wageningen, Netherlands) was used to analyze changes in gait parameters associated with motor dysfunction in NMO mouse model on 3 and 5 days after induction of NMO model, according to a previously described protocol [32].

All the above behavioral experiments were quantified by raters blinded to treatment assignment to assess the effect of BTK inhibitor on motor deficit.

#### Magnetic resonance imaging scanning

Brain MRI was performed using 9.4-T small-animal MRI to evaluate lesion volumes in alive mice on day 5 after NMO–IgG injection. T2-weighted images were acquired with follows parameters to detect lesion: repetition time (TR) = 2500 ms; effective echo time (TE) = 33 ms; resolutio*n* = 0.078 × 0.078 mm, number of signal averages = 2, and imaging time = 2 min 40 s. A total of 24 slices were acquired with thickness = 0.5 mm; field of view = 2.0 cm × 2.0 cm; and matrix 256 × 256. The MRI data were analyzed using ImageJ and MRIcroN4.0 software.

#### Immunofluorescence staining

Immunofluorescence staining was performed to characterize pathologically morphological and functional changes of glial cells**.** Mice brains were removed and postfixed with 4% PFA. After gradient dehydration in sucrose solution, tissues were embedded in OCT compound (Tissue-Tek, Torrance, CA, USA), and cryosectioned at 7 μm (Leica CM1850, Leica Instruments) for immunofluorescence study. The sections were incubated with primary antibodies: rabbit anti-206 (1:200, Immunoway, YT5640), rabbit anti-CD86 (1:500, Abcam, ab239075), mouse monoclonal anti-GFAP (1:1000, Abcam, ab4648), rabbit anti-AQP4 (1:100, Millipore, ABN411), rat anti-C3 (1:200, Abcam, ab11862), rabbit anti-S100A10 (1:500, Immunoway, YT4198), rabbit anti-Phospho-Btk (Tyr344) (1:200, Thermo Fisher Scientific, PA5-105620), rabbit anti-Btk Polyclonal antibody (1:200, Thermo Fisher Scientific, PA5-27392). Sections were then incubated with appropriate secondary antibody. Fluorescent signals were examined using a Leica TCS SP5 laser-scanning. Cells of interest were counted, and fluorescence signal intensity was quantified using ImageJ software (NIH). Data were analyzed in three sections of the same animal.

### Transmission electron microscopy (EM)

The animals used for the transmission electron microscopy (EM) were perfused 5 days after intracerebral injection with 20 ml of PBS, followed by 30 ml of fixative (2.5% glutaraldehyde, 4% PFA in phosphate buffer; 0.1 M) and washed with phosphate buffer pbs, and staying overnight in PBS, and then fixed in Osmium tetroxide (2%). Osmium tetroxide was diluted with double distilled water. Osmium-fixed samples were dehydrated and embedded in epon resin, as described previously [33]. Ultrathin sections were stained with uranyl acetate and Lead citrate, and samples were examined with Transmission electron microscopy HITACHI HT7800 under acceleration voltage of 60 kV.

### MBP staining and intracellular cytokine staining

Brain single cell suspension was prepared referring to a previous study [34].

Microglia were stained for fluorochrome-conjugated monoclonal antibodies after incubated with Fc-block (BD) at 4 ℃. The monoclonal antibodies are as follows: mouse CD45 (103114, Biolegend), mouse CD11b (101205, Biolegend). Intracellular MBP staining was performed according to a previous study [34]. Microglia were incubated with anti-MBP (Abcam) or IgG2a isotype control, for 1 h at room temp after fixation and permeabilization with an Intracellular Fixation and Permeabilization kit (554714, BD), followed by 30 min incubation with anti-mouse IgG Alexa Fluor 647 (ThermoFisher).

For intracellular cytokine staining, cells were re-stimulated with a cell stimulation cocktail (plus protein transport inhibitors) (00-4975-93, eBioscience; containing PMA and ionomycin in the presence of a protein transport inhibitor cocktail containing Brefeldin A and Monensin). Then, microglia were stained for fluorochrome-conjugated monoclonal antibodies after incubated with Fc-block (BD) at 4 ℃. The monoclonal antibodies are as follows: mouse CD45 (103114, Biolegend), mouse CD11b (101205, Biolegend), mouse IL-6 (504507, Biolegend) and mouse TNF-α (506327, Biolegend). Intracellular cytokine staining was performed using an Intracellular Fixation and Permeabilization kit (554714, BD), according to the manufacturer’s instructions.

### Preparation of SMART-seq2 RNA-seq libraries, sequencing, and data analysis

Brain single cell suspension was prepared and stained with PE-cy7-CD45 (103114, Biolegend), FITC–CD11b (101205, Biolegend) as well as LIVE/DEAD Fixable Aqua Dead Cell Stain to exclude dead cells. The detailed gating strategy is as follows. First, single events were gated on the FSC-H versus FSC-W dot plots. And then, microglia were gated on CD45^int^CD11b^+^ after excluding dead cells. Appropriate isotype controls were used in all steps to confirm staining specificity. Microglia were isolated by flow cytometry sorting (FACS Aria III, BD Biosciences, San Jose, CA, USA) and the sorted cells were lysed in RLT-buffer (74004, QIAGEN) for RNA extraction. A total amount of 1 μg RNA per sample was used as input material for the RNA sample preparations. Sequencing libraries were generated using NEBNext® UltraTM RNA Library Prep Kit for Illumina® (NEB, USA) following manufacturer’s recommendations and index codes were added to attribute sequences to each sample. Raw data (raw reads) of fastq format were first processed through in-house perl scripts. In this step, clean data (clean reads) were obtained by removing reads containing adapter, reads containing ploy-N and low-quality reads from raw data. At the same time, Q20, Q30 and GC content the clean data were calculated. All the downstream analyses were based on the clean data with high quality. After cluster generation, the library preparations were sequenced on an Illumina Novaseq platform and 150 bp paired-end reads were generated. Feature Counts v1.5.0-p3 was used to determine the number of reads mapped to each gene, after which each gene’s FPKM (expected number of fragments per kilobase of transcript sequence per million base pairs sequenced) was calculated based on the length of the gene and the number of reads mapped to the gene.

### Statistical analysis

All analyses were performed in the R Bioconductor suite. Data were measurements from distinct biological replicate samples and are presented as mean ± SEM, statistical significance was determined using two-tailed Student’s t tests or Mann–Whitney tests. In the case of three or more data sets, means were compared with two-way ANOVA with Bonferroni correction or Kruskal–Wallis with a Dunn’s multiple comparison test or mix-effects two-way ANOVA. Differences were considered significant for *p* < 0.05. Statistical analyses were performed using GraphPad Prism (Version 8; La Jolla, CA, USA). All statistical tests were two-tailed.

## Results

### Patient characteristics

As presented in Table 1, all 28 patients fulfilled criteria for seropositive NMOSD [24]. Twenty-six of 28 (93%) were female, and patients had a wide age distribution at disease onset as well as widely varying disease durations. Typically, patients had persistent serum AQP4–IgG levels despite several years of immunotherapy, a well-recognized feature of NMOSD [35].

### scRNA-seq of B cells identified fourteen distinct cell clusters and compartmental specificity

**Fig. 1:**
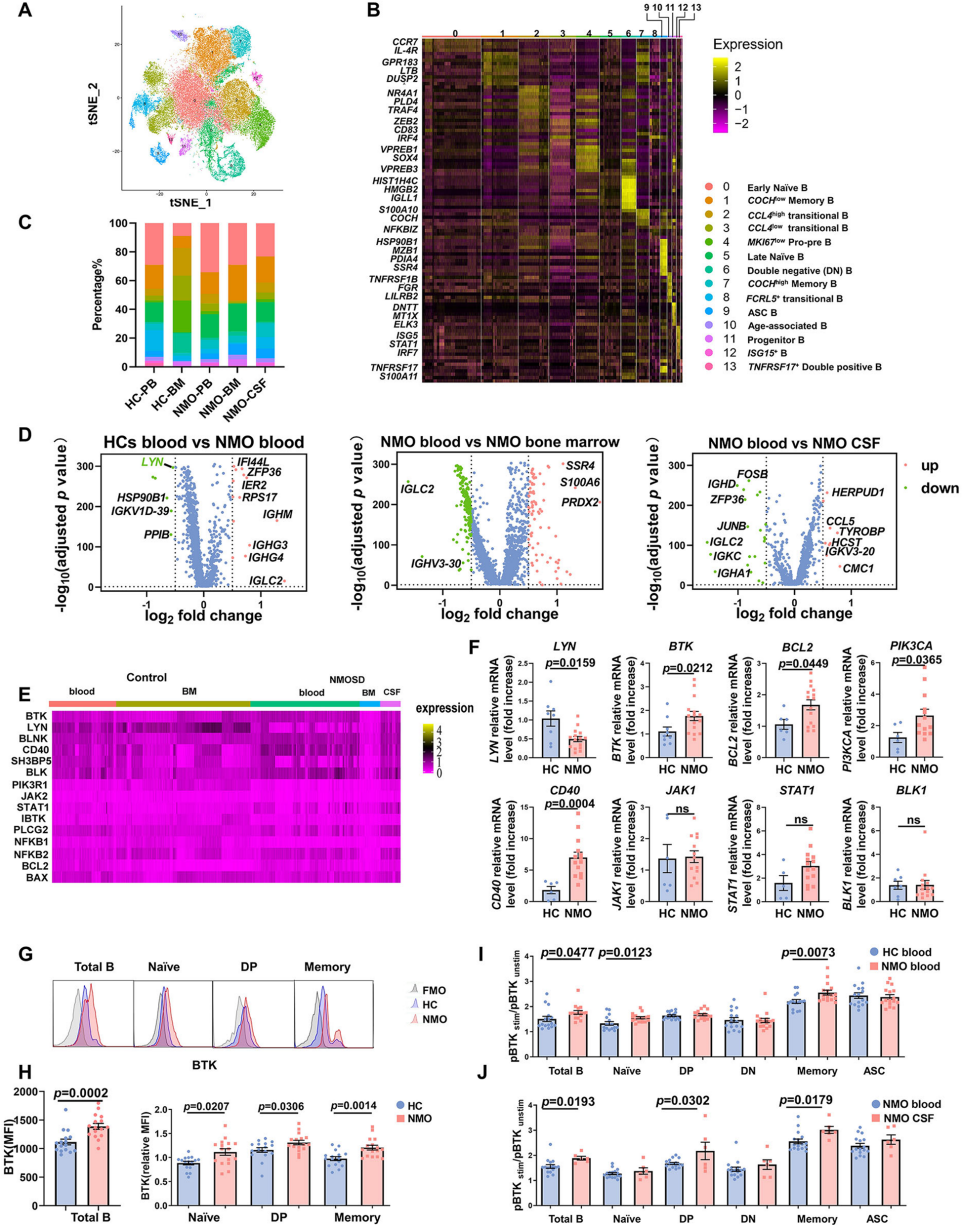
Single-cell RNA-sequencing and flow cytometry characterization of BTK expression in NMOSD. **A** Visualization of clustering by t-distributed stochastic neighbor embedding (t-SNE) plot of all B cells obtained from blood, CSF and bone marrow. **B** Heat map of scaled gene expression for top ten differentially expressed genes identifying each cluster, with selected genes listed. **C** Average proportion of each cell type derived from healthy controls (HCs, blood, *n* = 3; bone marrow, *n* = 2) and NMOSD (blood, *n* = 6; bone marrow, *n* = 2; CSF, *n* = 4) samples. **D** Volcano plot of differential expression genes of B cells between blood of patients with NMOSD and HCs or CSF/bone marrow from patients with NMOSD. Green, downregulated; red, upregulated. **E** Heatmap of differential expression genes (DEGs) involving *BTK*-related genes in B cells from different sources. **F** Expression of BCR signaling-related genes mRNA in peripheral blood B cells from HCs and patients with NMOSD was analyzed using real-time PCR. *BTK* (HCs*, n* = 9 and NMOSD, *n* = 18); *LYN* (HCs, *n* = 8 and NMOSD, *n* = 15); *BCL2, PIK3CA, CD40, JAK1* (HCs, *n* = 6 and NMOSD, *n* = 15), *STAT1* (HCs, *n* = 5 and NMOSD, *n* = 15) and *BLK1* (HCs, *n* = 7 and NMOSD, *n* = 15). **G** Representative flow plots show BTK protein expression in a NMOSD patient, a healthy control, and a fluorescence minus one (FMO). **H** BTK protein expression was measured by intracellular flow cytometry in total B cells and B-cell subpopulations in HCs and NMOSD patients, normalized to BTK expression in total B cells of HCs, which was set to 1.0. (*n* = 16 per group). Phospho-BTK (pBTK) expression in total B cells and the indicated B-cell subpopulations that were either left unstimulated (pBTK_unstim_) or were stimulated (pBTK_stim_) for 30 s with anti-IgM (20 μg/ml) in blood of NMOSD patients and HCs** (I)** (*n* = 16 per group) or in blood and CSF from NMOSD patients (**J)** (blood, *n* = 16; CSF, *n* = 6). Data are displayed as means ± SEM. Statistical analysis was conducted by Student’s t test

For transcriptomic characterization of B cells in NMOSD, we obtained 56,486 single B cells from patients with NMOSD during acute attacks and HCs; 28,344 cells were from the blood, 24,867 cells from the bone marrow, and 3,275 cells from the CSF. Our previous study identified four major subpopulations of B cells across these tissues by use of Scanpy 1.4.3 for clustering. These subpopulations are: naïve B cells, memory B cells, age-associated B cells, and antibody-secreting cells. In NMOSD B cells tend to become hyperreactive according to type I interferon pathway [13]. The current study revealed 14 novel subpopulations of B cells, including a continuum of phenotypic states from CD34^+^ hematopoietic stem and progenitor cells to terminal plasma cells in NMOSD. A cluster 11 expressing high levels of *CD34* represents progenitor B cells (Fig. 1A). Cluster 4 with high levels of *VPREB3* is designated as pro-pre B cells. Based on the levels of *IGHD*, *CD27*, and *CD38*, we identified *CCL4*^high^ transitional B cells (cluster 2), *CCL4*^low^ transitional B cells (cluster 3), and *FCRL5*^+^ transitional B cells (cluster 8). Several key canonical B-cell subtypes were also identified, in particular: naïve B cells (cluster 0 and cluster 5), memory B cells (cluster 1 and cluster 7), antibody secreting cells (ASCs) (cluster 9), double negative (DN) B cells (cluster 6), and *TNFRSF17*^+^ double positive (DP) B cells (cluster 13). Consistent with our prior research, age associated B cells (cluster 10) had high levels of *ITGAX* (*CD11C*), *CREM*, *TNFRSF1B*, and *TBX21* (*T-BET*) (Fig. 1B)**.** Moreover, we could detect cluster 12 as *ISG*^high^ B cells which had upregulated expression of *ISG15*, similar to the findings in lupus nephritis [36].

To determine the relationship between cell types, we plotted the data across the three tissue types in patients with NMOSD and HCs. We found that progenitor and pro-pre B cells also circulated in the CSF of NMOSD patients, although their functions in the brain remain unknown. These NMOSD patients also exhibited a higher percentage of memory B cells and ASCs in the CSF compared with peripheral blood of the same group, displaying a specific mature profile (Fig. 1C).

Next, we used Reactome enrichment pathway analyses to characterize each compartment (blood, CSF, and bone marrow). In line with our previous study, activity of type I interferon signaling was enhanced in all B cells populations in NMOSD blood (Additional file 1: Fig. S1A) [13]. Compared with CSF B cells, NMOSD blood B cells displayed significant protein methylation, GTP hydrolysis and joining of the 60S ribosomal subunit, and IFN α/β signaling **(**Additional file 1: Fig. S1B). B cells from NMOSD bone marrow showed significant enrichment in pathways related to ER stress response, metabolism of proteins and nucleotides, as well as detoxification of ROS **(**Additional file 1: Fig. S1C).

### Bruton’s tyrosine kinase activity is upregulated in blood and CSF B cells in patients with NMOSD

Next, we sought to comprehensively analyze differentially expressed genes in B cells across blood, CSF, and bone marrow in patients with NMOSD. We focused on B-cell receptor (BCR), as sustained BCR signaling is required for the survival of B cells in the periphery through the induction of NF-κB-dependent and/or other pro-survival signaling [37, 38]. We found a significant decrease in the expression of *LYN* (Lck/Yes-Related Novel Protein Tyrosine Kinase) in NMOSD blood B cells compared with that in HCs blood B cells (Fig. 1D). Heatmap of BCR signaling-related genes in B cells showed that the expression of BTK fluctuates between different sources. Specifically, *BTK* expressing cells tended to be more frequent in NMOSD blood than in HCs blood, although the difference is not statistically significant; conversely, *LYN* expression was reduced in NMOSD blood (Fig. 1E). Considering the small sample size of RNA-seq, which may influence the accuracy, we applied RT-PCR to detect mRNA of these genes mentioned above in magnetic-activated cell-sorted B cells from the blood samples of HCs and patients with NMOSD. We found that *BTK*, *BCL2*, *PIK3CA* and *CD40* were up-regulated and mRNA of *LYN* was decreased in NMOSD (Fig.1F).

We compared BTK expression in B cells from treatment-naive patients with AQP4–IgG ( +) NMOSD and HCs using flow cytometry staining. The results revealed that the expression of BTK in total blood B cells was significantly higher in patients with NMOSD and BTK was differentially expressed between different B-cell subsets. Naïve B cells, double positive B cells, and class-switched memory B cells demonstrated higher expression of BTK protein in NMOSD compared with HCs (Fig. 1G, H).

To determine BTK functional activity, we stimulated B cells ex vivo with F(ab′)_2_ anti-human IgM (20 μg/ml) for 30 s and quantified phosphorylated BTK (pBTK, Tyr223 phosphorylation) by intracellular flow cytometry. Since the level of pBTK fluctuated among patients, we compared the ratio of pBTK _stim_/pBTK_unstim_ between HCs and NMOSD patients and found that this ratio was significantly increased in total blood B-cell naïve B cells and class-switched memory B cells in NMOSD (Fig. 1I). We further compared pBTK expression in NMOSD B cells from blood and CSF and found that CSF B cells had higher ratio of pBTK _stim_/pBTK_unstim_ compared with that in blood B cells. The increase was characteristic of CSF double positive B cells and class-switched memory B cells (Fig. 1J).

### Inhibition of BTK attenuated B-cell activation in patients with NMOSD

**Fig. 2:**
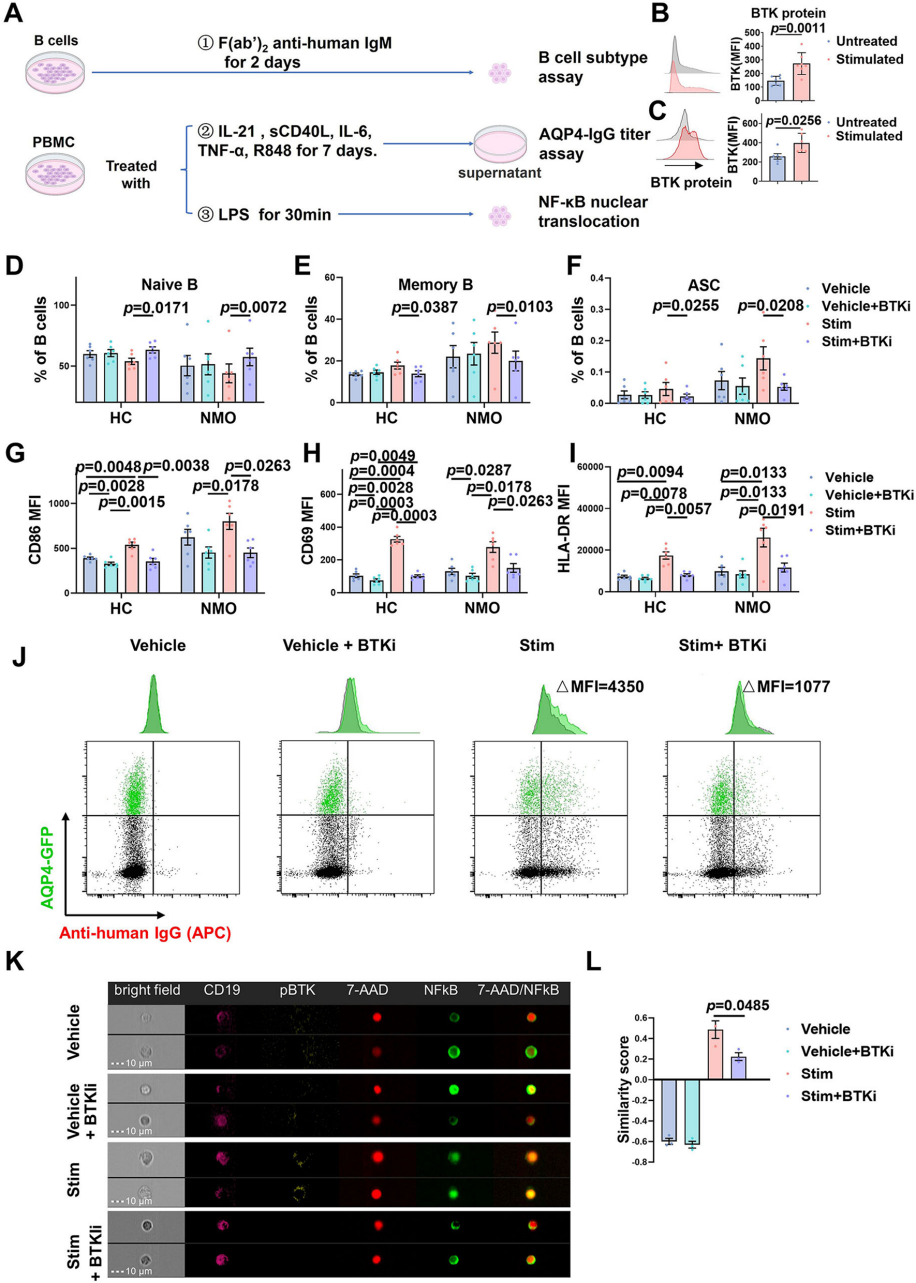
BTK inhibition limits B-cell activation and differentiation in NMOSD.** A** Experimental design to examine the effect of BTK inhibition on B-cell subtypes, production of AQP4 antibody and intracellular signaling pathway under different stimulants. **B**, **C** Representative graph and quantitative analysis of intracellular BTK expression from a NMOSD patient. (**B**: sorted B cells after 2 day culture of either untreated or activated with F(ab′)_2_ anti-human IgM; **C**: PBMC after 7 day culture of either untreated or activated). **D–I** Quantitative analysis of the proportions of naive B cells (**D**) switched memory B cells (**E**) and ASCs (**F**) and the expression of CD86 (**G**), CD69 (**H**), HLA-DR (**I**) in magnetic-activated cell-sorted B cells from healthy controls or NMOSD patients after 2 days of stimulation with F(ab’)_2_ anti-human IgM (10 μg/ml) in the absence or presence of BTKi (zanubrutinib, 10 nM) (*n* = 6 per group). **J** Binding of patient IgG from culture supernatants to live HEK-293 T cells co-expressing AQP4 and green fluorescent protein (GFP) from a single plasmid. Quantitative titres defined as the difference in mean fluorescence intensity (MFI) between the GFP-expressing and GFP-negative cell populations (expressed as △MFI). **K** NF-κB nuclear translocation staining showed B cells failed to translocate NF-κB from cytoplasm to the nucleus upon stimulation after administration of zanubrutinib. **L** Analyses of similarity scores showed NF-κB translocation to the nucleus of B cells. Representative of 3 experiments. The spatial relationship between the NF-κB and nuclear images was measured using the ‘Similarity’ feature in the IDEAS® software. Data are displayed as means ± SEM. **D**–**I** Statistical analysis was conducted by Mix-effects two-way ANOVA, **L** Repeated measure two-way ANOVA

Considering the good performance of BTK inhibitor in autoimmune arthritis [39] and multiple sclerosis [40], we chose to focus on the effect of BTK inhibitor on NMOSD. The experimental protocol to test the effects of BTK inhibition on B-cell activation is shown in Fig. 2A. Using magnetic-activated cell-sorted B cells, we found that BTK expression was up-regulated after stimulated by F(ab′)_2_ anti-human IgM for 2 days (Fig. 2B). BTK inhibitor (BTKi) zanubrutinib reduced the generation of class-switched memory B cells and ASCs after F(ab′)_2_ anti-human IgM simulation (Fig. 2D–F). Moreover, zanubrutinib strongly limited BCR-mediated (F(ab′)_2_ anti-human IgM) induction of the activation markers CD86 (Fig. 2G), CD69 (Fig. 2H) and HLA-DR (Fig. 2I), but also somewhat diminished expression of these n markers under basal culture conditions (Vehicle + BTKi), although the changes were not statistically significant, suggesting that the effects of zanubrutinib on human B-cell responses may not be limited to consequences of BCR-mediated signaling.

We established in vitro blood PBMCs culture conditions which promote pathological antibody production to investigate the effects of zanubrutinib on the generation of these autoantibodies. Expression of BTK was up-regulated after cultured in this condition for 7 days (Fig. 2C). Flow cytometry showed that zanubrutinib treatment effectively reduced AQP4–IgG secretion in patients with NMOSD (Fig. 2J). However, the AQP4 antibody level may vary between individuals. This result may predict the potential effects of BTKi on some patients with NMOSD in their generation of pathogenic autoantibodies,

To identify the association between zanubrutinib and canonical NF-κB signaling, we monitored NF-κB translocation and analyzed similarity scores following B-cell activation by LPS for 30 min with or without BTK inhibition. Absence of pBTK and the presence of NF-κB in the cytoplasm of the CD19^+^ B cells in the unstimulated groups are shown in Fig. 2K, L. In the absence of zanubrutinib in activated B cells NF-κB was translocated from the cytoplasm to the nucleus; following zanubrutinib treatment, however, most of NF-κB remained in the cytoplasm. B cells without stimulation had a similarity score of negative value: the scores were − 0.60 in vehicle and − 0.63 in vehicle + BTKi group, indicating the presence of NF-κB in their cytosol (Fig. 2L). However, the score increased to + 0.49 following activation in the absence of zanubrutinib. Consequently, after zanubrutinib treatment, the similarity score was reduced to + 0.22, consistent with the result shown in Fig. 2K. Thus, we show that NF-κB is a downstream pathway following BTK activation in B cells and that these cells become unresponsive to activation following zanubrutinib treatment.

### Inhibition of BTK reduced pathology in NMO mouse model

**Fig. 3:**
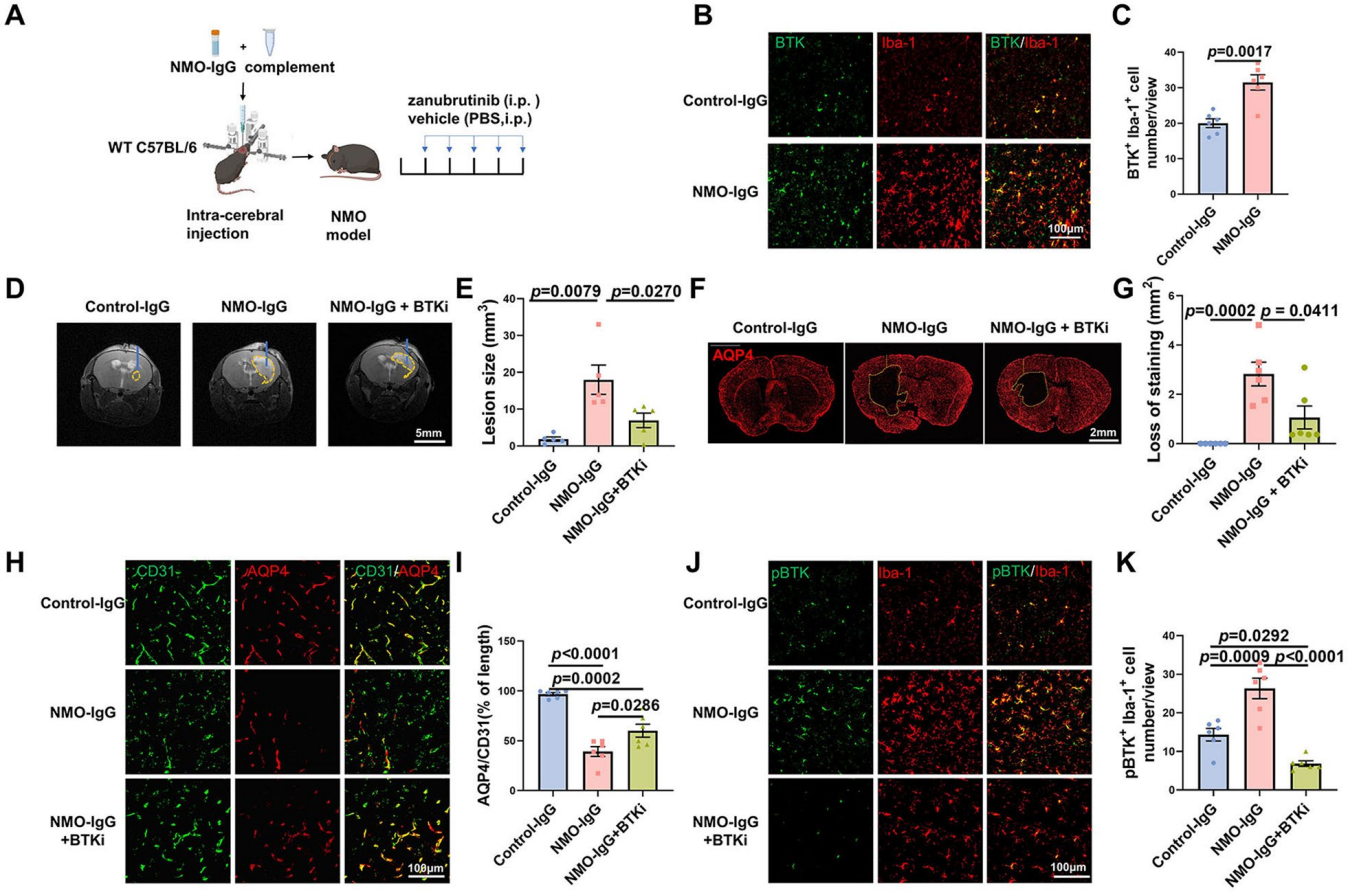
NMO–IgG intracerebral injection induced AQP4 loss and enhanced expression of BTK and pBTK in microglia. **A** Experimental NMO mice were induced with NMO–IgG and complement. The mice were administered zanubrutinib (5, 10, 20 mg/kg) for 5 days. **B** Immunofluorescent staining of BTK protein expression in the brains of mice either treated with control–IgG or NMO–IgG. **C** Numbers of BTK^+^ Iba-1^+^ microglia were analyzed in control–IgG recipient mice (control–IgG) and NMO–IgG mice. **D** MRI presentation in different groups. T2-weighted images showed lesion areas dotted with yellow dashed lines, The blue lines represent the needle tract. **E** Quantification of the lesion volume induced by NMO–IgG after receiving BTKi treatment or not. **F** Immunofluorescent staining of AQP4 at 5 days after injection. The yellow lines delimit the lesion with AQP4 loss. Scale bar is 200 μm. **G** Quantification of the lesion size induced by NMO–IgG after receiving BTKi treatment or not. **H** Colocalization of astrocytic and endothelial cells in the absence or presence of zanubrutinib. Astrocytes were stained with AQP4 (red), and endothelial cells were stained with CD31 (green). **I** Quantification of relative length of CD31^+^ parenchymal blood vessels covered by AQP4 around NMO–IgG induced lesion (*n* = 6 per group). **J** Immunofluorescence staining of phosphorylated BTK (Y223) (pBTK) expressed on microglia in the absence or presence of zanubrutinib. **K** Numbers of pBTK^+^ Iba-1^+^ microglia were analyzed in control–IgG recipient mice (control–IgG) and NMO–IgG mice in the absence (NMO–IgG) or presence (NMO–IgG + BTKi) of zanubrutinib (20 mg/kg) (*n* = 6 per group). The NMO–IgG + BTKi represented NMO–IgG recipient mice with the treatment of 20 mg/kg zanubrutinib, for no significant statistical differences were found when the zanubrutinib dosages were 5 or 10 mg/kg (data not shown). Scale bars, 100 μm. Data are displayed as means ± SEM. Statistical analysis was conducted by one-way ANOVA or Student’s t test

Since validating the beneficial effect of BTK inhibition in repressing B-cell activity, we asked whether BTK inhibition with zanubrutinib alleviates disease severity in NMO mice, since up-regulated expression of BTK can be seen in NMO mouse model (Fig. 3B). Zanubrutinib was administered for five consecutive days after establishment of the NMO mouse model (Fig. 3A). After treatment, motor functions were evaluated. In the rotarod test, the average latencies to fall in NMO mice were significantly shorter than the control–IgG recipient (control–IgG) group. Zanubrutinib treatment prolonged the average latencies in a dose-dependent manner on day 3 or day 5, compared to NMO mice without treatment (Additional file 1: Fig. S2A). Subsequently, we evaluated gait strength in the mice. A similar trend could be observed in the zanubrutinib treatment group (Additional file 1: Fig. S2B–D). Collectively, NMO mice displayed motor deficits in the rotarod performance and gait experiment, and zanubrutinib treatment contributed to the improvement of functional disability.

On day 5, 9.4-T MRI was performed to visualize lesions (T2) and evaluate their volume. We found a significant reduction in lesion volume in the high dosage of BTKi (20 mg/kg zanubrutinib)-treated NMO mice compared with the NMO–IgG group (Fig. 3D, E), but no significant statistical differences were found when the dosages were 5 or 10 mg/kg zanubrutinib (data not shown). This result was further confirmed by immunofluorescence staining for AQP4 (Fig. 3F, G). The result of immunofluorescence staining for AQP4 and CD31 indicated that AQP4 expression in astrocytic end-feet was co-localized with the vasculature marker CD31 in the control–IgG recipient mice brain. In NMO brains, AQP4 loss was significant, while CD31 expression was relatively preserved. The relative length of CD31^+^ vasculature covered by AQP4 was higher in zanubrutinib-treated (only in 20 mg/kg zanubrutinib but not in 5 or 10 mg/kg zanubrutinib) NMO mice compared with untreated NMO mice (Fig. 3H, I). We found pBTK expression was increased in reactive microglia in NMO mice, and zanubrutinib significantly reduced the level of microglial pBTK (Fig. 3J, K).

**Fig. 4:**
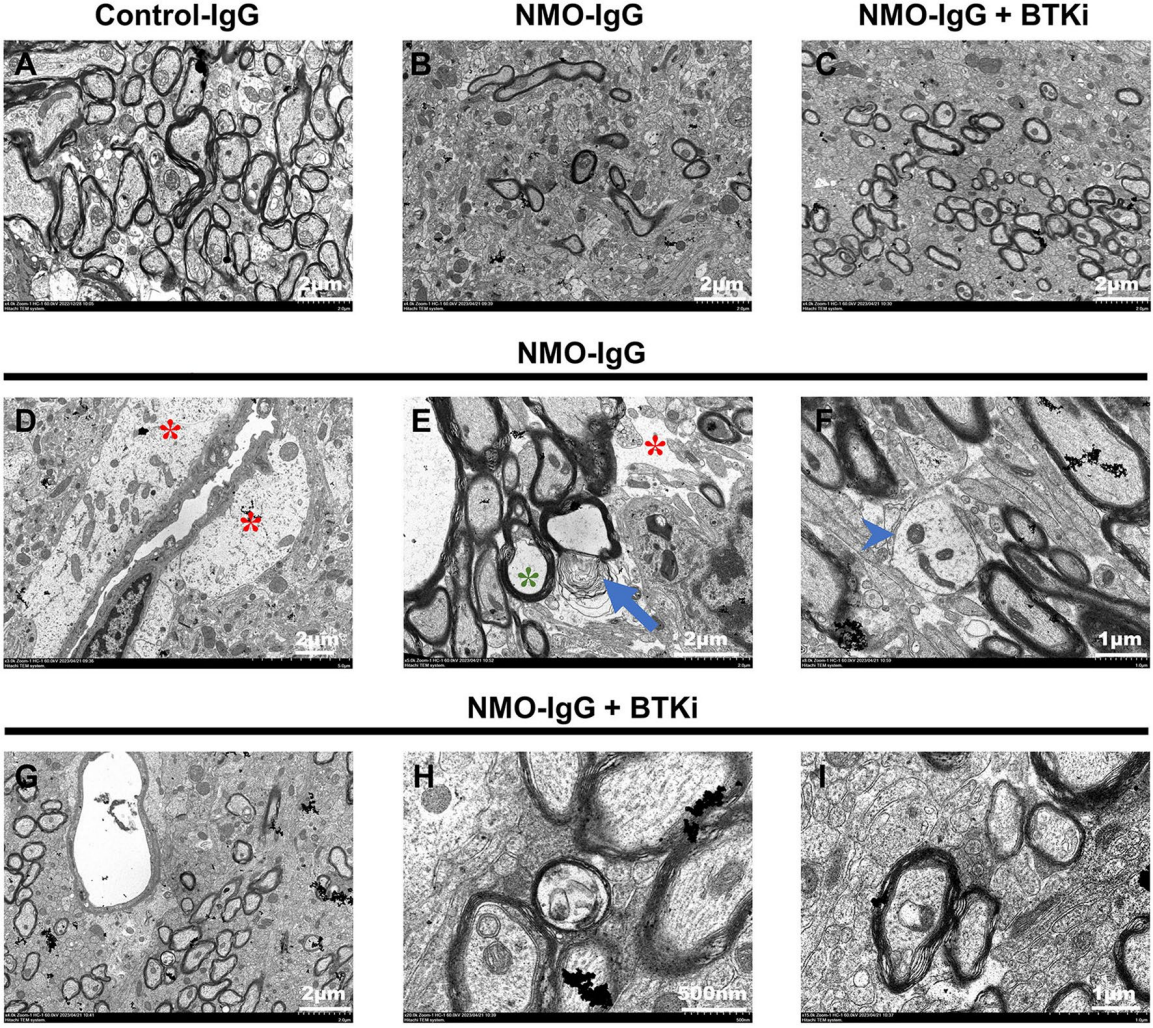
Electron microscopy analysis of axonal pathology in experimental NMO lesions. **A**–**C** Representative electron microscopy images of experimental NMO lesion in the brain of Control mice, NMO mice model either untreated, or after 5 days of BTK inhibitor treatment. **D**, **G** Edema around vessels *(red asterisk)* can be seen in the images of untreated NMO model but not BTK inhibitor treated group. **E**, **H** Multifocally, a breakdown of myelin characterized by separation and/or vesiculation of myelin lamellae *(arrows)*, myelin sheath edema *(green asterisk)*, and an extracellular edema *(red asterisk)* is more significant in untreated NMO model compared with BTK inhibitor treated group. **F**, **I** High-resolution transmission EM image of untreated NMO model shows naked axons *(arrowheads)* and microtubule disorganization. Well-arranged, densely packed microtubules are visible in NMO model with BTKi treatment. The NMO–IgG + BTKi represented NMO–IgG recipient mice with the treatment of 20 mg/kg zanubrutinib

We examined the morphology of NMO lesions in different groups by serial transmission electron microscopy. More demyelinated axons can be seen in the NMO–IgG recipient compared with that of BTKi treated NMO–IgG group (Fig. 4A–C). In particular, NMO–IgG injection increased the permeability of the blood–brain barrier (BBB) and instigated BBB leakage (Fig. 4D) NMO–IgG injection also caused a breakdown of myelin characterized by separation and/or vesiculation of myelin lamellae, myelin sheath edema (Fig. 4E) and cytoskeletal remodeling marked by sparse and disorganized microtubules (Fig. 4F). BTKi treatment partially mitigated the impairments mentioned above (Fig. 4G–I).

### Inhibition of BTK alleviated reactive microgliosis and astrogliosis and microglia–astrocyte pathological interactions

**Fig. 5:**
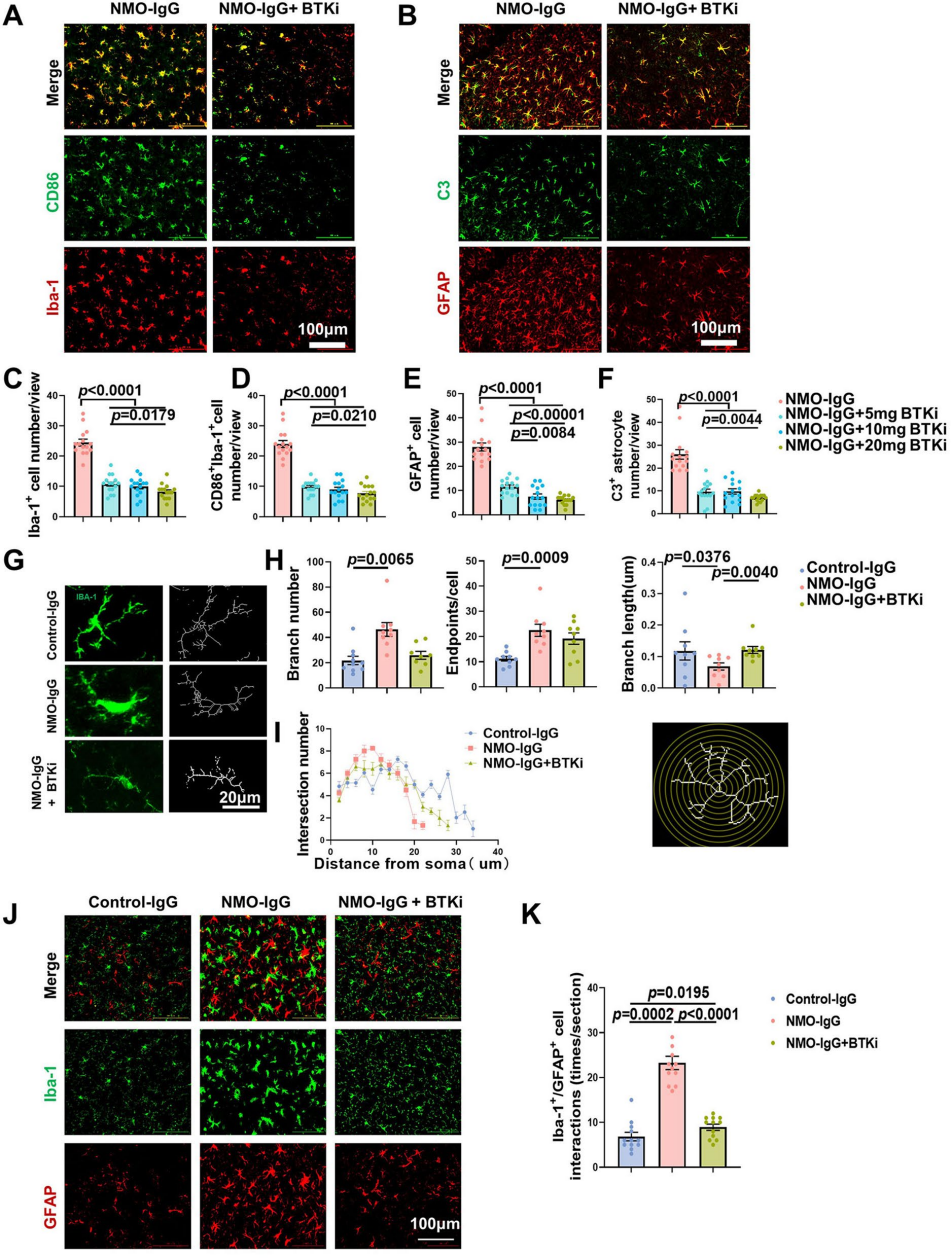
BTK inhibition attenuated microglia and astrocyte recativity as well as their interaction in NMO mice. Immunofluorescent staining of microglia (**A**) and astrocytes (**B**) in the absence or presence of zanubrutinib (20 mg/kg). Activated microglia were stained with CD86 (green) and Iba1 (red). Reactive astrocytes were immune-stained with GFAP (green) and complement C3 (red). Quantification of the numbers of Iba1^+^ microglia (**C**), Iba1^+^ CD86^+^ microglia (**D**), GFAP^+^ astrocytes (**E**) and GFAP^+^ C3^+^ astrocytes (**F**) in the absence or presence of zanubrutinib treatment at stepwise doses (5, 10 or 20 mg/kg). *n* = 6 mice per group. Scale bars, 100 μm. **G**–**I** Morphological analysis of microglia in control–IgG recipient mice (control–IgG) and NMO–IgG mice in the absence (NMO–IgG) or presence (NMO–IgG + BTKi) of zanubrutinib (20 mg/kg), *n* = 6 mice per group. **G** The morphology and skeleton of microglia, Scale bars, 20 μm. **H** Quantification analyses of process length, endpoints number, and branch number in microglia. **I** Sholl analysis of microglia on day 5 after NMO–IgG mice model was induced. Data are displayed as means ± SEM. Statistical analysis was conducted by one-way ANOVA. **J** Interaction between Iba1^+^ microglia (green) and GFAP^+^ astrocytes (red) are inferred from the enlargement and overlapping of cells and their processes in dual immunostaining. Zanubrutinib was administered at a dose of 20 mg/kg. **K** Quantification of Iba1^+^ microglia and GFAP^+^ astrocytes interaction in control–IgG recipient mice (control–IgG) and NMO–IgG mice in the absence (NMO–IgG) or presence (NMO–IgG + BTKi) of zanubrutinib (20 mg/kg). *n* = 6 mice per group (3 sections/mouse)

The numbers of Iba-1^+^ microglia and Iba-1^+^ CD 86^+^ microglia were reduced after zanubrutinib treatment (Fig. 5A, C and D). The morphological analysis of astrocytes also showed that BTK inhibition with zanubrutinib reduced the numbers of GFAP^+^ and GFAP^+^C3^+^ reactive astrocytes (Fig. 5B, E and F). Skeleton analysis showed that following AQP4–IgG injection microglia in NMO mice underwent a striking morphological change, from surveilling ramified phenotype to a reactive bushy phenotype. However, zanubrutinib treatment limited microglial reactivity as indicated by an increase in branch length (Fig. 5G, andH). Sholl analysis showed a reduction in microglial process complexity in NMO mice, whereas zanubrutinib recovered microglial morphology (Fig. 5I). These data indicated that BTK inhibition with zanubrutinib attenuated microgliosis in NMO mice.

Astrocyte–microglia interaction drives evolution of NMO lesion [17], so we assessed the interaction between these glial cells in the absence or presence of BTK inhibitor. Coalescence of astrocytes and microglia was enhanced more than fourfold following NMO–IgG injection and this was significantly reduced after the administration of zanubrutinib (Fig. 5J, K). Thus, the reduced crosstalk of astrocytes and microglia may be, at least in part, responsible for the mitigated pathology.

### BTK inhibition enhanced the phagocytosis but reduced intracranial injection-induced increases in pro-inflammatory cytokine levels in microglia

Using a novel assay developed to quantify phagocytosed intracellular myelin basic protein (MBP) in microglia by flow cytometry we found that phagocytosis of myelin was increased after BTK inhibitor treatment, as indicated by the differences in the percentage of MBP^+^ microglia NMO–IgG versus NMO–IgG + BTKi mice (Additional file 1: Fig. S3A). In addition, the proinflammatory properties of microglia was attenuated after BTK inhibition (Additional file 1: Fig. S3B, C).

### RNA sequencing with smart-seq2 reveals transcriptome changes of microglia in NMO lesions after BTK inhibition

**Fig. 6:**
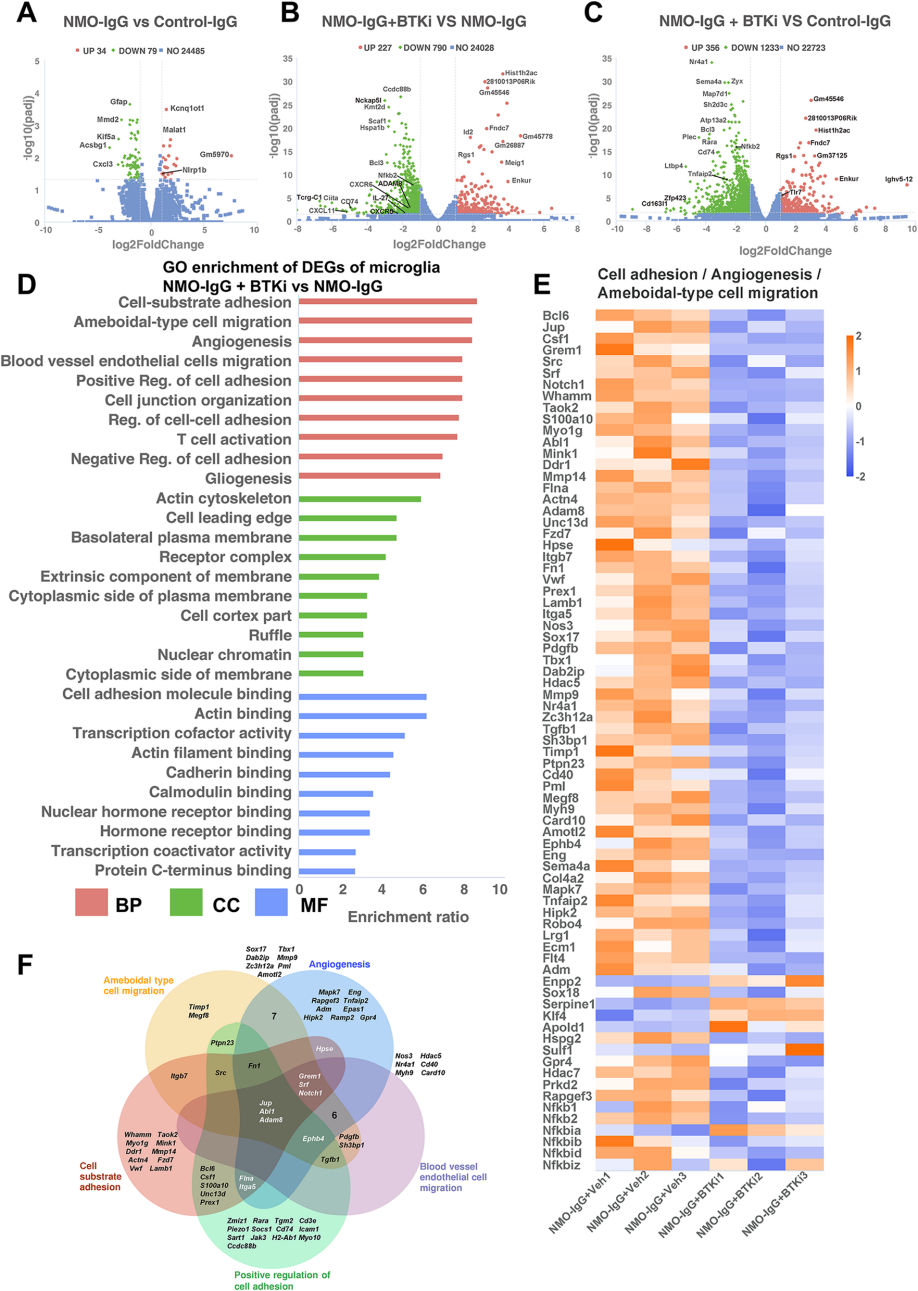
RNA sequencing reveals microglia changes in response to NMO–IgG injection and BTKi treatment. **A–C** Volcano plot of differential expression genes (DEGs) of microglia between NMO–IgG recipient and control–IgG recipients (**A**), between NMO–IgG recipient with BTKi treatment or not (**B**), between NMO–IgG recipient with BTKi treatment and control–IgG recipients (**C**). **D** GO pathway analysis for biological processes (red), molecular functions (green) and cellular components (blue) enrichment of DEGs of microglia from untreated NMO models and NMO models with BTKi treatment. **E** Heatmap showing specific genes involved in the biological processes ‘Cell adhesion’, angiogenesis and ‘ameboidal-type cell migration’ (scale = Z scores of FPKM values). **F** Venn diagram showing overlap between genes in the top 5 biological process. The NMO–IgG + BTKi represented NMO–IgG recipient mice with the treatment of 20 mg/kg zanubrutinib

To better understand pathophysiological contribution of microglia, RNA sequencing was used. We first filtered for genes that are expressed at an appreciable level (FPKM > 1). Differentially expressed genes were identified using the DESeq2 package in R Studio (v3.6.3). Specific filtering criteria (adjusted *p* < 0.05, |log2 fold-change|> 1) were employed to identify genes that were significantly upregulated or downregulated in NMO–IgG microglia relative to control–IgG microglia and NMO–IgG + BTKi microglia relative to NMO–IgG, control–IgG microglia. The result showed that 34 genes were upregulated and 79 genes were downregulated in NMO–IgG versus control–IgG microglia (Fig. 6A); 227 and 790 genes were upregulated and downregulated, respectively, in NMO–IgG + BTKi microglia versus NMO–IgG microglia (Fig. 6B); 356 and 1233 genes were upregulated and downregulated in NMO–IgG + BTKi microglia versus control–IgG microglia (Fig. 6C).

Functional gene set enrichment analysis to identify gene ontology (GO) terms was performed using Web-based Gene Set Analysis Toolkit. Go analysis confirmed that the major biological processes of NMO–IgG + BTKi microglia relative to NMO–IgG were: cell-substrate adhesion, ameboidal-type cell migration, angiogenesis, blood vessel endothelial cell migration, positive regulation of cell adhesion (Fig. 6D, E). The main molecular functions affected by BTKi were actin binding, transcription cofactor activity, actin filament binding, cadherin binding, calmodulin binding (Fig. 6D). In line with this, the main cellular components affected by BTKi treatment were actin cytoskeleton, cell leading edge, basolateral plasma membrane, receptor complex, extrinsic component of membrane (Fig. 6D). Next, a Venn diagram was created to visualize genes in the top 5 biological processes that were significantly affected by BTKi treatment (Fig. 6F), and found 3 genes (Jup, Abl1 and Adam8) were involved in all of the top 5 biological processes.

## Discussion

BTK is a cytoplasmic tyrosine kinase expressed mainly in B cells and myeloid cells [41]. Since tyrosine kinases play crucial roles in cellular proliferation, growth, differentiation and other life-sustaining processes, while both B cells and myeloid cells (particularly microglia) are important drivers of MS progression [42], BTK inhibitors were suggested as potential candidates for the treatment of MS and being investigated in phase 2/3 clinical trials. In a phase 2 trial of relapsing–remitting multiple sclerosis (RRMS), treatment with covalent inhibitor of BTK evobrutinib significantly decreased lesions through 24 weeks of observation compared to placebo. Up to date, phase 3 clinical trials are underway to evaluate the efficacy in annualized relapse rates (ARR) of evobrutinib or tolebrutinib compared to teriflunomide through a 96-week treatment period [43–45]. Cumulative evidence indicates that B cells contribute to the pathogenesis of both MS and NMOSD in the periphery. Not only B cells produce antibodies, but also cellular properties of B cells such as antigen presentation and cytokine production shape the response of T cells and myeloid cells and drive inflammation [46]. This resulted in exploring the utility of targeting B cells to contain disease activity in these disorders.

In this study we demonstrated the heterogeneity of B cells in NMOSD and detected their differential signaling pathways across the blood, CSF, and bone marrow from patients. BCR activation-related molecules *LYN* and *BTK* were identified. Prior studies have shown that upon B-cell activation, LYN mediates inhibitory signaling pathways by BCRs, whereas defects in LYN are associated with human lupus [47, 48]. The LYN kinase is known to inhibit BTK-dependent pathways in B cells [49]. Comparison of *LYN*^−/−^ mice with wild-type animals also identified BTK inhibition as a potential therapy target [50, 51], thus providing new insights into BTK signaling in NMOSD. In patients with NMOSD, we confirmed increased expression of BTK and pBTK in B cells. BTK inhibitor zanubrutinib suppressed B-cell maturation and AQP4–IgG production. In the CNS, BTK was expressed mainly in microglia. We used a NMO mouse model to demonstrate the reactive microgliosis and astrogliosis. Zanubrutinib protected against pathological microglia–astrocyte interactions and attenuated CNS damage in NMO mice. The dual roles in both B cells and microglia suggest that BTK inhibition warrants further studies in patients with NMOSD.

The BTK plays a crucial role in setting the threshold for B-cell activation and counterselection of autoreactive B cells [27]. In several autoimmune diseases, BTK activity is enhanced in peripheral blood B cells [26]. In the present study, we show that BTK is differentially expressed in B-cell subpopulations in NMOSD. Expression of BTK was significantly increased in naïve B cells, CD27^+^IgD^+^ non-switched memory B cells, and switched memory B cells, whereas pBTK was predominantly increased in naïve and switched memory B cells. These findings suggest a role for BTK upregulation in the early activation of B cells and in terminal stage of B-cell differentiation and maturation. As a key mediator of BCR signaling in B cells, BTK may promote an autoinflammatory loop [52]. Modulation of BTK activity has the potential to affect multiple inflammatory responses caused by B cells. Zanubrutinib, a highly selective inhibitor of BTK, could inhibit malignant B-cell proliferation and was approved for the treatment of mantle cell lymphoma [53]. In this study, upon BCR stimulation in vitro, the frequency of naïve B cells decreased but memory B cells and ASCs increased, indicating that B cells tended to differentiate into mature B cells after stimulation. Zanubrutinib contributed to restoring the balance of B-cell subpopulations. Furthermore, our results showed that zanubrutinib reduced the expression of activation markers CD86, CD69, and HLA-DR on B cells. In line with ibrutinib, the first generation BTK inhibitor, which blocks plasmablast generation and production of autoantibodies in systemic lupus erythematosus [54–56], zanubrutinib reduced AQP4–IgG production in vitro.

B cells not only contribute to chronic inflammatory conditions as source of antibody-secreting plasma cells, but also may exert regulatory effects. Some researchers identified B-cell-provided interleukin (IL)-10 as key factor in controlling pro-inflammatory activity of peripheral myeloid cells as well as microglia [57]. On the other hand, monocytes can also have an impact on B cells, as shown in a study that TLR7-mediated B-cell differentiation was strongly dependent on the cross-talk between B cells and monocytes [58]. Recent research demonstrated that potential cross-talk between disease-relevant human B-cell subsets and both resident CNS microglia and infiltrating macrophages may propagate CNS-compartmentalized inflammation and injury associated with MS disease progression [59].

All the mentioned above represents an attractive therapeutic target for BTKi that modulate responses of both B cells and microglia, because BTK is also expressed in microglia and is related to microglial function [34]. Inhibition of BTK activity attenuated microglia proliferative response induced by myelin oligodendrocyte glycoprotein antibody injection [34]. BTK was involved in regulating microglial phagocytosis and the uptake of synaptic structures in Alzheimer’s disease [60]. Accumulating evidence also show that inhibition of BTK favors remyelination and may be a promising new therapeutic strategy to promote remyelination by targeting microglia [61]. In this study, we found that zanubrutinib inhibited reactive microgliosis in NMO mice models. A recent study demonstrated that astrocyte–microglia interaction may drive the evolution of the NMO lesions [17]. Our data demonstrated that zanubrutinib reduced pathological interactions between the microglia and astrocytes following induction of NMO pathology. We also found that phagocytosis of myelin was increased after BTK inhibitor treatment, which may account for the mitigated demyelination visualised by electron microscope. Phagocytosis is considered one of the prerequisites for remyelination in MS. If phagocytosis is hampered, excess myelin debris leads to the reduction of Pdgfr-α and Igf-1 signals and stimulates Ifn-γ secretion. Both of them may impair oligodendrocyte progenitor cells recruitment, proliferation, and maturation, resulting in impaired remyelination [62–64]. In addition, the proinflammatory properties of microglia were attenuated after BTK inhibition. To explore the possible mechanism BTK inhibitor action on microglia, we analyzed our sequencing data on microglia from NMO–IgG recipient with or without BTK inhibitor treatment and revealed that the main molecular functions affected by BTKi were related to cytoskeleton. A previous study demonstrated that BTK is a major signaling molecule responsible for activating actin remodeling for B-cell spreading and BCR clustering [65]. Researches on Chronic lymphocytic leukemia (CLL) also confirmed correlation between treatment with BTK inhibitors and cell motility [66, 67]. From the studies mentioned above and the data provided by SMART seq, we may suggest that the effect of BTK inhibitor on microglia is achieved, at least partially, through the actin cytoskeleton. We further identified several candidate genes that may drive proinflammatory properties of microglia, including *IL-27*, *CXCL11* and *ADAM8*. The levels of IL-27 were elevated in MS brain tissues [68, 69] and it promoted the expression of CXCL9, CXCL10, and ICAM-1 in astrocytes [70]. CXCL11 is a chemokine regulating leucocyte migration, inflammation, and immune function [71]. ADAM8 is a disintegrin matrix metalloproteinase that disrupts the extracellular matrix by cleaving chondroitin sulphate proteoglycan and promoting barrier disruption [72], while ADAM8 is activated in microglia during neurodegeneration [73]. We suggest that the downregulation of these molecules in microglia may modify interaction between reactive microglia and astrocytes.

BTK inhibitors, such as zanubrutinib, may have therapeutic effect in NMOSD by modulating responses of both B cells and microglia, in contrast to anti-CD20 or anti-CD19 monoclonal antibodies that deplete B cells. Zanubrutinib is the second generation of BTK inhibitors with high selectivity. Compared with the first generation ibrutinib, zanubrutinib has much higher IC_50_ and no inhibition on kinase activities of ITK, JAK3, HER2, FGR, LCK, CSK, FYN, HCK, in other words, zanubrutinib was shown to be a highly specific BTK inhibitor, without off-target effects at relevant concentrations [74]. Zanubrutinib also has the capacity to penetrate the blood brain barrier [75]. Currently, a phase 2 trial is ongoing to evaluate the efficacy of zanubrutinib in patients with NMOSD (ClinicalTrials.gov, NCT05356858).

There are several limitations of our study. First, the transcriptomic analysis does not fully elucidate the regulatory effect of different B-cell subtypes in the CSF and blood. Moreover, analysis of a larger group of patients is needed to draw significant conclusions about the relationship between BTK protein, pBTK, and disease severity in NMOSD. In addition, we did not evaluate the role of B cells in our NMO mouse model, because this passive transfer model produced major pathological lesions in the CNS by AQP4–IgG and complement, otherwise it did not directly result in significant B-cell dynamics both in the CNS and periphery. In fact, no model has achieved spontaneous AQP4 autoimmunity with pathology in optic nerve and spinal cord [76]. Although zanubrutinib is able to penetrate the blood–brain barrier to enter the CNS [75], the central effects of zanubrutinib on pathological T cells in the CNS remains unknown.

## Conclusion

We demonstrated that inhibition of BTK in B cells and reactive microglia ameliorates neuroinflammation in NMOSD. This study provides pre-clinical evidence for BTK inhibition as a promising treatment option without B-cell depletion for NMOSD.

### Supplementary Information

**Additional file 1: Figure S1.** Pathway analysis of B cells across the compartments. **A** Reactome biological process enrichment of DEGs of blood B cells from NMOSD and HCs. **B**, **C** Reactome biological process enrichment of DEGs of B cells from different tissue of NMOSD patients. **Figure S2.** Zanubrutinib mitigated the motor impairment induced by NMO–IgG intrathecal infusion**. A **Rotarod tests showed motor impairment (measured as fall latency) with injection of NMO–IgG (*n =* 6 for each group), but BTK inhibitor–zanubrutinib can attenuated the impairment and this effect seemed dose-dependent. **B **Gait illustrated by representative paw print images after 3 days of NMO–IgG injection with or without zanubrutinib.** C**, **D **Stride length of NMO–IgG recipients (*n =* 6 for each group) was shorter than that of wild type (WT) (*n =* 6), indicating significant gait impairment, but zanubrutinib can attenuated the impairment and this effect seemed dose-dependent. Right forelimb, RF; Right hindlimb, RH; Left forelimb, LF; Left hindlimb LH. **Figure S3.** Zanubrutinib enhanced the phagocytosis of microglia but reduced intracranial injection-induced increases in pro-inflammatory cytokine levels in microglial cells. Quantification of percent myelin basic protein (MBP) positive (**A**), IL-6 positive (**B**) and TNF-α positive (**C**) microglia from NMO model with or without BTK inhibitor treatment. Data analyzed by unpaired t test with Welch's correction from representative experiment. (*n* = 3).

## Data Availability

The data supporting the findings of this study are available within the article and its additional file.

## Abbreviations

AQP4–IgG: Aquaporin-4 antibody
ASC: Antibody-secreting cell
BTK: Bruton’s tyrosine kinase
BTKi: BTK inhibitor
DEGs: Differential expression genes
HCs: Healthy controls
IPA: Ingenuity pathway analysis
IPND: The International Panel for NMO Diagnosis
LYN: Lck/Yes-related novel protein tyrosine kinase
MFI: Median fluorescence intensity
MS: Multiple sclerosis
NMOSD: Neuromyelitis optica spectrum disorder
PBMC: Peripheral blood mononuclear cell
pBTK: Phosphorylated BTK
scRNA-seq: Single-cell RNA-sequencing
UMI: Unique molecular identifier

## Availability of data and materials

The data sets, including flow cytometry data used and analyzed during the current study, are available from the corresponding author upon reasonable request.
