# Supplementary material for: Bruton’s tyrosine kinase-bearing B cells and microglia in neuromyelitis optica spectrum disorder
Source: J Neuroinflammation. 2023 Dec 21;20:309. doi: 10.1186/s12974-023-02997-2 (PMC10740299; doi:10.1186/s12974-023-02997-2)
Supplement: Supplementary file 1 — Additional file 1: Figure S1. Pathway analysis of B cells across the compartments. A Reactome biological process enrichment of DEGs of blood B cells from NMOSD and HCs. B, C Reactome biological process enrichment of DEGs of B cells from different tissue of NMOSD patients. Figure S2. Zanubrutinib mitigated the motor impairment induced by NMO–IgG intrathecal infusion. A Rotarod tests showed motor impairment (measured as fall latency) with injection of NMO–IgG (n = 6 for each group), but BTK inhibitor–zanubrutinib can attenuated the impairment and this effect seemed dose-dependent. B Gait illustrated by representative paw print images after 3 days of NMO–IgG injection with or without zanubrutinib. C, D Stride length of NMO–IgG recipients (n = 6 for each group) was shorter than that of wild type (WT) (n = 6), indicating significant gait impairment, but zanubrutinib can attenuated the impairment and this effect seemed dose-dependent. Right forelimb, RF; Right hindlimb, RH; Left forelimb, LF; Left hindlimb LH. Figure S3. Zanubrutinib enhanced the phagocytosis of microglia but reduced intracranial injection-induced increases in pro-inflammatory cytokine levels in microglial cells. Quantification of percent myelin basic protein (MBP) positive (A), IL-6 positive (B) and TNF-α positive (C) microglia from NMO model with or without BTK inhibitor treatment. Data analyzed by unpaired t test with Welch's correction from representative experiment. (n = 3). [file 12974_2023_2997_MOESM1_ESM.docx]

**Figure S1**

**
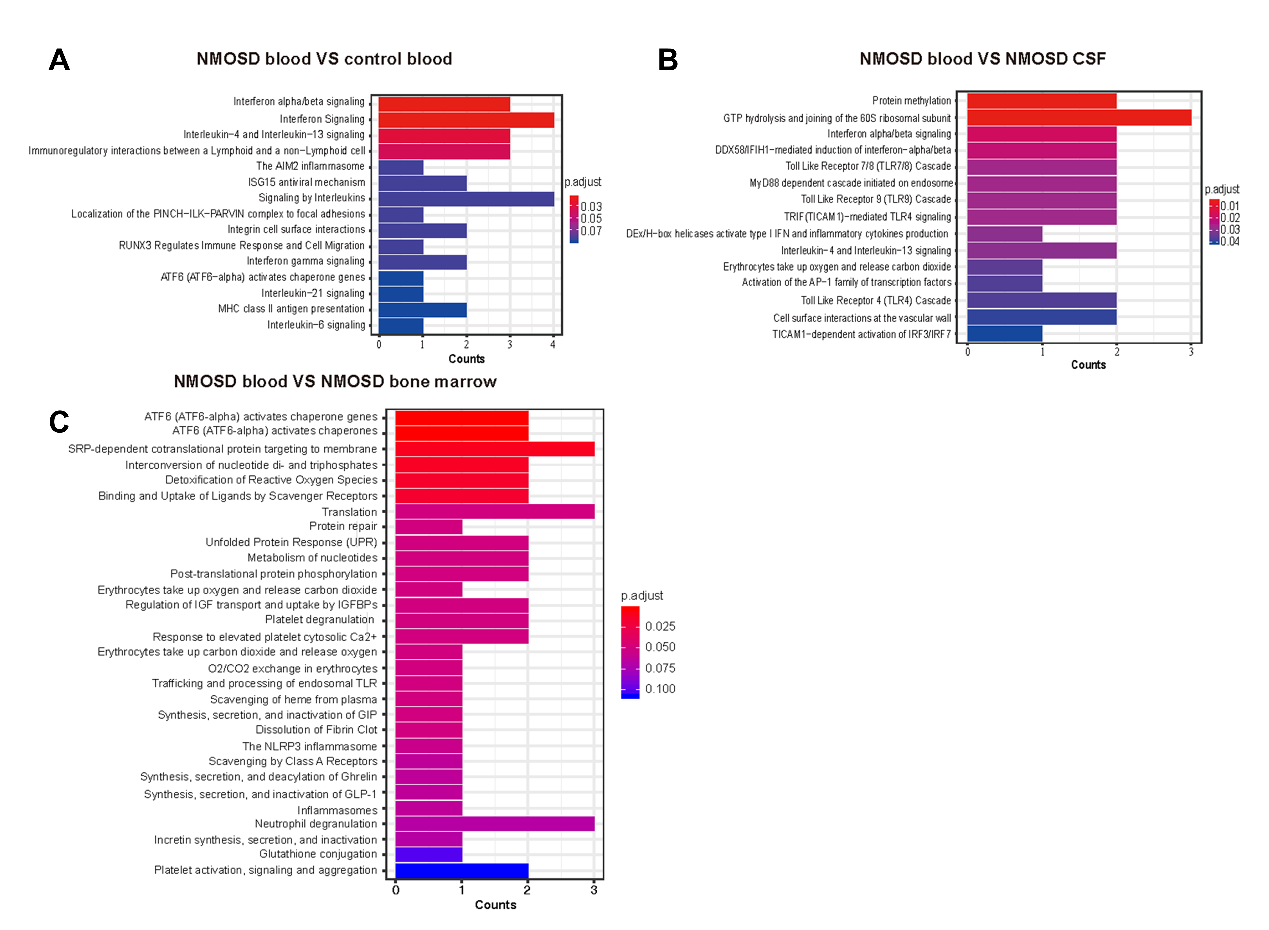
**

**Figure S1 Pathway analysis of B cells across the compartments. (A)** Reactome biological process enrichment of DEGs of blood B cells from NMOSD and HCs. (**B, C)** Reactome biological process enrichment of DEGs of B cells from different tissue of NMOSD patients.

**Figure S2**

**
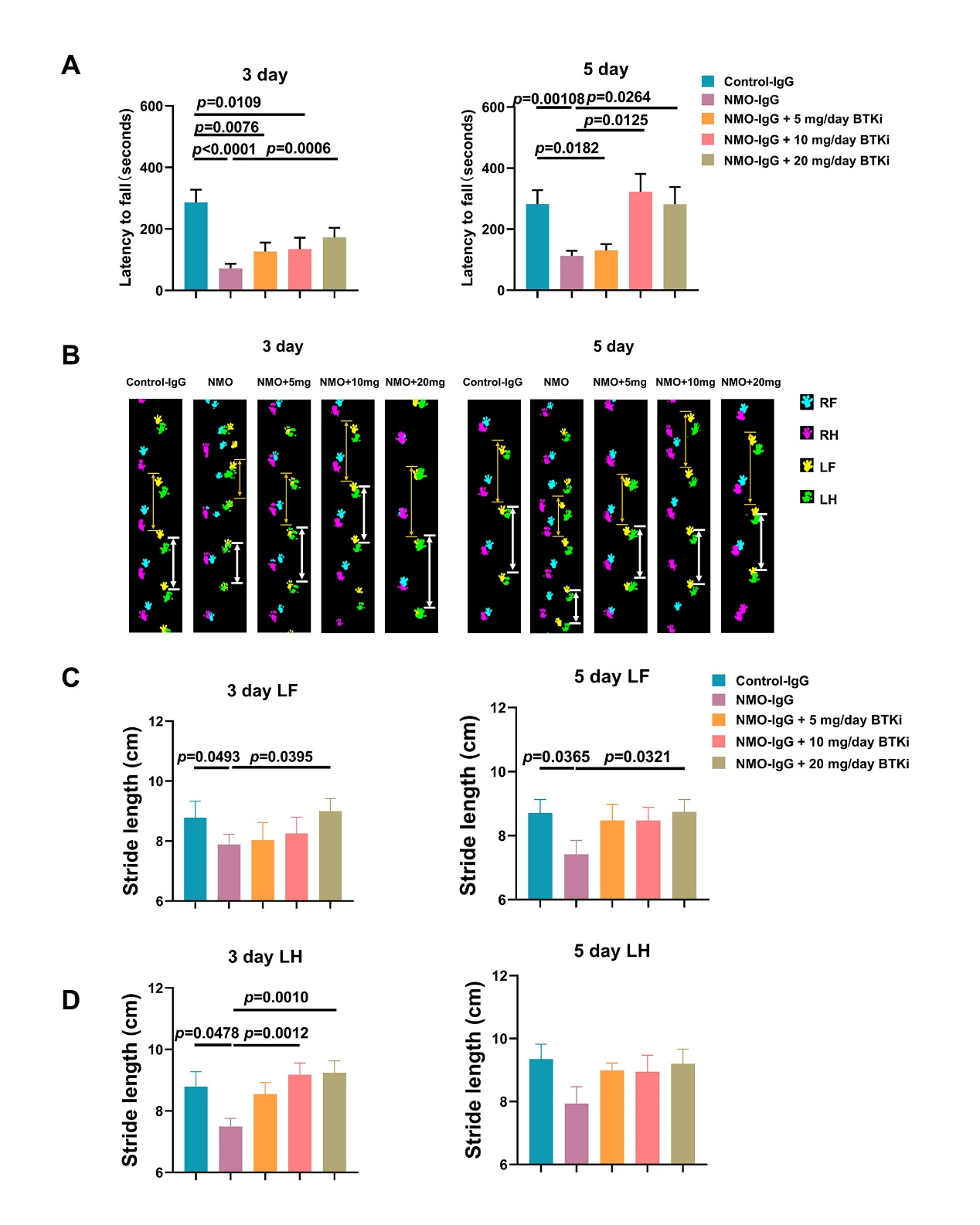
**

**Figure S2 Zanubrutinib mitigated the motor impairment induced by NMO-IgG intrathecal infusion A** Rotarod tests showed motor impairment (measured as fall latency) with injection of NMO-IgG (*n =* 6 for each group), but BTK inhibitor-zanubrutinib can attenuated the impairment and this effect seemed dose-dependent. **B** Gait illustrated by representative paw print images after 3 days of NMO-IgG injection with or without zanubrutinib. **C**, **D** Stride length of NMO-IgG recipients (*n =* 6 for each group) was shorter than that of wild type (WT) (*n =* 6), indicating significant gait impairment，but zanubrutinib can attenuated the impairment and this effect seemed dose-dependent. Right forelimb, RF; Right hindlimb, RH; Left forelimb, LF; Left hindlimb LH.

**Figure S3**

**
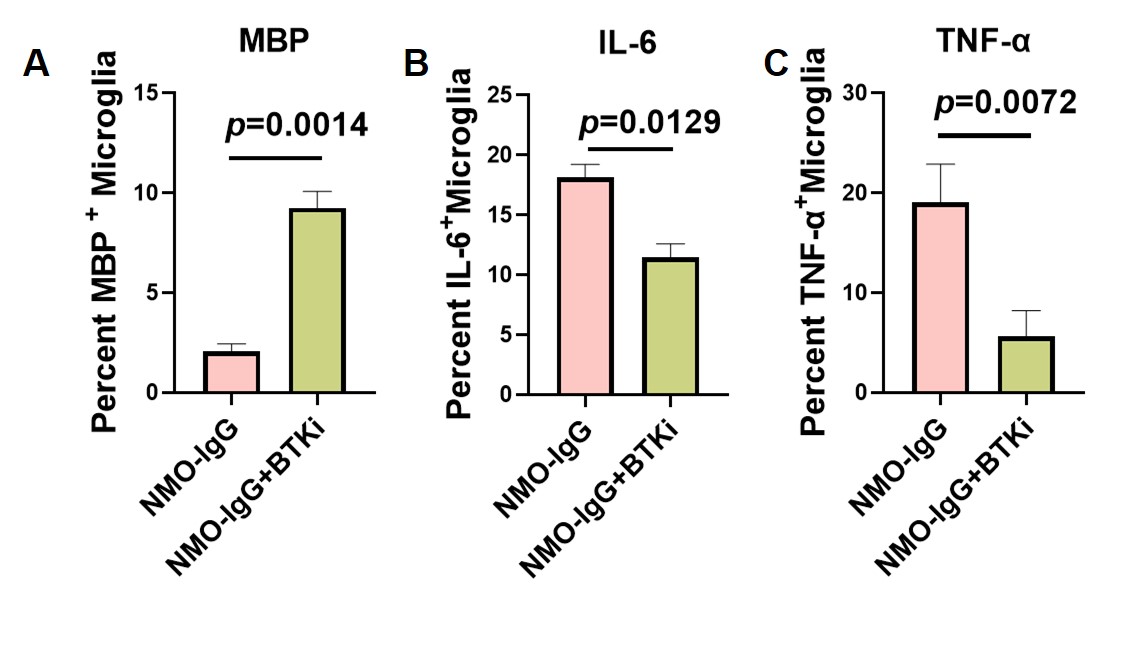
**

**Figure S3 Zanubrutinib enhanced the phagocytosis of microglia but reduced intracranial injection-induced increases in pro-inflammatory cytokine levels in microglial cells**

Quantification of percent myelin basic protein (MBP) positive (A), IL-6 positive (B) and TNF-α positive (C) microglia from NMO model with or without BTK inhibitor treatment. Data analyzed by unpaired t test with Welch's correction from representative experiment. (n = 3)

**Methods**

**MBP staining.** Microglia were first incubated with Fc-block at 4oC and then stained with fluorochrome conjugated antibody for CD11b, CD45 as well as 7-AAD to gate on live cells. After surface staining, Microglia were fixed with 2% PFA. For intracellular MBP staining, cells were permeabilized in biolegend 1x permeabilization buffer and then incubated with anti-MBP or IgG2a isotype control for 1 hr at room temp, followed by 30 min incubation with anti-rat IgG. The stained samples were assessed by flow cytometry using a FACS Aria III flow cytometer (BD Biosciences, San Jose, CA, USA). The results were analyzed using FlowJo software (version 10).
